# Increase in the Number of Medical Schools in the United States and Its Potential Adverse Ramifications for Urology Residency Applicants

**DOI:** 10.7759/cureus.80508

**Published:** 2025-03-13

**Authors:** Umberto M Donato, Dominique M Ebedes, David Nelwan, Ahmad Imam, Trushar Patel

**Affiliations:** 1 Health Outcomes and Behavior Lab, Moffitt Cancer Center, Tampa, USA; 2 Pediatric Oncology, Tampa General Hospital, Tampa, USA; 3 Radiology, Moffitt Cancer Center, Tampa, USA; 4 Pediatric Hematology Oncology, University of South Florida Morsani College of Medicine, Tampa, USA; 5 Ophthalmology, University of South Florida Morsani College of Medicine, Tampa, USA; 6 Urology, University of South Florida Morsani College of Medicine, Tampa, USA

**Keywords:** accreditation council for graduate medical education (acgme), residency application process, resident mentor, undergraduate and graduate medical education, urology application, urology education, urology match, urology residencies, urology tips, urology training

## Abstract

Introduction: This study assesses the unbalanced expansion of medical education institutions in the United States regarding the growth of urology residency programs. We assessed the prevalence of medical schools offering urology residencies to identify potential barriers for students pursuing a urology residency.

Materials and methods: Data concerning year of establishment (YOE), number of urology residency positions, American Urological Association (AUA) section, and National Institutes of Health (NIH) funding were collated from online resources, including the Association of American Medical Colleges. This information was stratified based on the YOE: pre-2000 (YOE <2000) and post-2000 (YOE ≥2000), and by the presence or absence of urology residencies.

Results: A total of 218 medical schools were identified, consisting of 159 Doctor of Medicine (159 N, 72.9%) and 59 Doctor of Osteopathic Medicine (59 N, 27.1%) schools, alongside 148 urology residencies (148 N, 67.9%). Since 2000, 76 new medical schools (76 N, 34.9%) have been established. The proportion of medical training sites with an associated urology residency has decreased from 79.6% (113/142) prior to the year 2000 to 67.9% (148/218) at present. Significant disparities in NIH funding were identified between medical programs established before and after 2000. Among the 204 medical schools (204 N, 93.6%), 143 (70%) were established before 2000, while 58 (28.4%) were established after. Schools with a YOE <2000 were substantially more likely to receive NIH funding (83% N, 118/143) compared to those with a YOE ≥2000 (28%, 16/58, p<0.001). Schools established before 2000 received significantly higher funding (p<0.001).

NIH funding distribution across the established AUA sections was also analyzed. Pearson's chi-square test showed no significant differences in funding receipt among sections (p=0.326). A detailed breakdown of funding percentages, mean amounts, and standard deviations for each section highlighted regional variations in funding allocation.

Conclusions: The observed disparity in the growth of medical schools compared to urology residency programs may represent a significant barrier for prospective urology residents. Given the crucial roles of research/mentorship in medical training, it is essential to develop and implement accessible programs at both the AUA section and national levels for aspiring students, especially in schools without a urology residency program.

## Introduction

In 1765, William Shippen Jr. and John Morgan established the first medical school in the United States (US) at the College of Philadelphia [1]. Approximately a century later, Sir William Osler developed the first formal residency program at Johns Hopkins University [2]. At this same institution in 1915, the first US urology residency program was established, marking a milestone that set the foundation for the future of urology training [3]. Since then, urology training programs have grown significantly, with 148 active urology residency programs in the US as of April 2024 [4].

According to the Council on Graduate Medical Education, the total number of residents in the US was projected to rise by 24% between 2000 and 2020. However, despite this increase, the Association of American Medical Colleges (AAMC) has predicted an 86,000 full-time physician shortage by 2025, with 20% of this shortage comprising surgical subspecialists [5,6].

To address projected physician shortages, medical schools have increased student admissions per class and established new medical schools [5]. Consequently, this growth has led to more applications for residency programs yearly. In 2022, urology programs recorded a historic high of 8.1 applications per interview [7]. The disproportionate growth of medical schools concerning urology residency programs has increased barriers for aspiring urologists.

Our study investigates the expansion of medical education institutions in the US concerning the growth of urology residency training programs. We analyzed the prevalence of schools with urology residency affiliations and assessed gaps/differences in medical school NIH funding across each AUA section to identify potential obstacles for medical students pursuing a urology residency across the US. Specifically, we found that schools without urology residency programs face unique challenges, and we recommend implementing urology-focused research fellowships and offering clinical rotations at hospitals with urology residency programs to enhance students' experience and competitiveness in the residency match.

## Materials and methods

Data sources

Data regarding total enrollment numbers, Doctor of Medicine (MD)/Doctor of Osteopathic Medicine (DO) accreditation, year of establishment (YOE), number of urology residency positions, and American Urological Association (AUA) section for various institutions was obtained using online resources including the AAMC, American Association of Colleges of Osteopathic Medicine, Doximity, and individual program websites. This data was then organized and stratified based on the YOE (either YOE <2000 or YOE ≥2000) and the presence or absence of a urology program.

Next, NIH funding data was extracted from the Blue Ridge Institute for Medical Research (BRIMR). This independent nonprofit organization ranks institutions, departments, and investigators annually and assesses the amount of NIH funding at each institution. These rankings are typically released shortly after the conclusion of the federal fiscal year and are accessible on the NIH's Research Portfolio Online Reporting Tool website. In our study, we used the data for the fiscal year 2022, which provides detailed information on funding that exceeded $36 billion, distributed across 65,307 extramural NIH grants or contracts awarded to more than 43,500 entities during that time frame [8]. We limited our study to the dataset pertinent to the 2022 calendar year and NIH awards assigned to SOMs. Our university’s institutional review board granted a waiver for this study’s approval as it utilized publicly available, de-identified data sourced from established databases such as the AAMC and BRIMR.

For the purposes of NIH funding analysis, the dataset encompassed a total of 204 schools. Independent residency programs without direct affiliations to a medical school were not included in this analysis, nor were any of the branches of medical schools with more than one location (i.e., if a given medical college had multiple branches, only the main campus was taken into consideration). These exclusions were necessary as the data for branch campuses was often not separately available, and the NIH funding was typically attributed to the main campus, ensuring consistency and accuracy in the analysis.

Statistical analysis

Receipt of NIH funding was compared by urology residency programs and MD/DO programs with the Pearson's chi-square test. The amount of funding was assessed using the Mann-Whitney U test. Statistical significance in the study was defined using p-values, with a threshold of p<0.05 indicative of a significant difference between groups. Percentage calculations of undergraduate medical training sites with a urology residency, comparing YOE <2000 to YOE >2000 and the previously mentioned analyses, were completed using SPSS Statistics version 29 (IBM Corp. Released 2023. IBM SPSS Statistics for Windows, Version 29.0.2.0 Armonk, NY: IBM Corp).

## Results

General description

**Figure 1:**
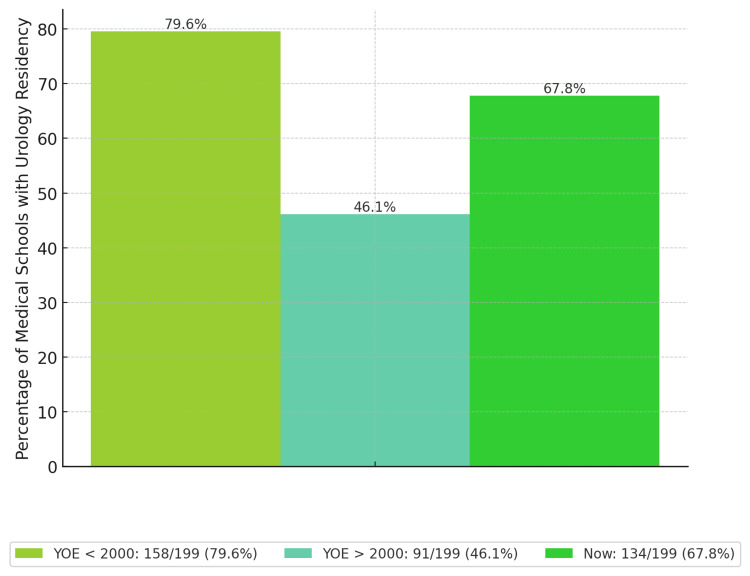
Percentage of medical schools with a urology residency by YOE YOE <2000: medical schools with YOE before 2000; YOE >2000: medical schools with YOE after 2000; Now: current medical schools offering urology residency YOE: year of establishment

Before 2000, there were 142 medical schools (126 MD, 16 DO), representing 65.1% of the total, with 113 accredited urology residencies (79.6%). Since 2000, an additional 76 undergraduate medical training sites have been established (33 MD, 43 DO), accounting for 34.9% of the total and 35 new urology residency programs. Currently, there are 218 undergraduate medical training sites (159 MD, 59 DO), comprising 100% of the total, and 148 urology residencies (67.9%). A comparison of the percentage of medical schools with urology residency programs, categorized by YOE (before and after 2000), revealed variations in growth (Figure 1).

Forty-seven (23%) institutions were classified as DO schools and 157 (77%) as MD schools. Among the schools, 107 (52.5%) had a urology residency program. On average, schools with a urology program offered 1.53 spots (standard deviation (SD): 1.62). A total of 134 schools (65.7%) received funding from the NIH. For those that secured funding, the average overall funding was $136,209,564, with an SD of $171,196,591 (Table 1). The YOE of the schools ranged from 1,765 to 2,022. The student enrollment varied, with numbers ranging from 75 to 1,535 students. On average, the enrollment across all schools was 625.8 students (SD: 257.8).

**Table 1:** Variations and averages of NIH funding of medical schools This table outlines NIH funding amounts reported by medical schools (MD/DO) stratified by YOE. YOE: year of establishment, NIH: National Institutes of Health, SD: standard deviation, MD: Doctor of Medicine, DO: Doctor of Osteopathic Medicine

YOE	N (no. of schools)	Mean NIH funding	SD	Median	Range
<2000	118 (88.1%)	$153,285,080.98	$175,494,266.739	$74,632,281.00	$57,194.00-$751,039,395
>2000	16 (11.9%)	$10,277,627.81	$20,751,220.548	$5,134,333.00	$591,645-$86,476,039
Total	134 (100%)	$136,209,564.19	$171,196,591.281	$63,143,817.50	$750,982,201

Data analysis

Schools with a urology residency were significantly associated with receiving funding from the NIH based on Pearson's chi-square test (χ² = 66.121, p<0.001). Among schools with a urology residency program, 92.5% received NIH funding (mean: $181,789,765, SD: $179,641,214), whereas 38% of schools without a urology residency received funding (mean: $12,191,033, SD: $17,034,665). MD programs were significantly more likely to receive NIH funding compared to DO programs (p<0.001), with 86.4% of MD programs receiving funding, while only 2.2% of DO programs received funding.

There were significant differences in NIH funding between medical programs established before and after 2000. Of 204 medical schools examined, 143 (70%) were established before 2000, while 58 (28.4%) were established after. Schools with YOE <2000 were much more likely to receive NIH funding, with 83% compared to 28% of YOE >2000 schools (p<0.001). Additionally, YOE <2000 schools received significantly higher funding (Table 1, p<0.001).

NIH funding distribution across established AUA sections was examined. Pearson's chi-square test revealed no significant difference in funding receipt by section (p=0.326). Detailed breakdowns of funding percentages, mean amounts, and SDs for each section were provided, highlighting variations in funding across different regions (Table 2).

**Table 2:** NIH funding by AUA section N: number of medical schools; p<0.326 for receipt of funding by section NIH: National Institutes of Health, AUA: American Urological Association, SD: standard deviation

Section	N	N (%) with funding	Amount (mean)	Amount (SD)
Mid-Atlantic	20	16 (80%)	$118,294,368	$180,368,120.51
New England	9	8 (89%)	$159,104,979	$165,374,898.99
New York	14	10 (71%)	$202,046,773	$191,783,322.76
North-Central	27	24 (89%)	$127,471,566	$137,478,397.01
Northeastern	9	8 (89%)	$118,508,424	$181,484,767.35
South-Central	25	23 (92%)	$102,547,022	$141,309,322.99
Southeastern	36	27 (75%)	$113,432,637	$158,175,607.46
Western	25	17 (68%)	$213,962,309.82	$239,017,736.21

## Discussion

This study evaluated the growth of medical schools relative to the growth rate of urology residency programs. Additionally, it explored the relationship between NIH funding and the presence or absence of urology residencies at medical schools. The findings revealed a notable decrease in the proportion of medical schools with urology residency programs. While 80% (113/142) of schools established before 2000 had an associated urology residency, only 46.1% (113/218) of schools founded after 2000 offered such programs. This discrepancy may stem from newer medical schools lacking the time and funding to establish residency programs. However, the disparity significantly changes medical students' access to urology exposure and mentorship.

The study also identified decreased levels of NIH funding at institutions without urology residency programs compared to those with such programs. Schools with urology residencies were more likely to receive NIH funding and received significantly larger amounts. Schools with urology residencies had a mean NIH funding of $181,789,765 (SD: $179,641,214), whereas schools without these residencies had a mean funding of $12,191,033 (SD: $17,034,665). As of 2018, the top 50 research-funded institutions accounted for 84% (84%) of all NIH funding, while the remaining institutions represented only 16% [9]. This disparity indicates that opportunities for students to build research portfolios are increasingly concentrated at well-funded institutions, particularly those with urology residency programs.

The type of medical school also influenced NIH funding. MD programs received more funding than DO programs, raising concerns about disparities in research opportunities for MD versus DO students. Among the AUA sections, the Western AUA section experienced the greatest percentage decrease in medical schools with urology residencies. Still, it also showed the largest mean and percentage increase in NIH funding over the past 20 years [9]. These trends demonstrate that, despite a decline in urology residency programs relative to medical school growth, research funding remains concentrated among a select few well-funded institutions [10]. Between 2009 and 2018, the mean increase in NIH funding for the top 10 funded institutions was $72.4 million, compared to just $17.8 million for institutions outside the top 10 [9].

The findings align with existing literature, highlighting declining urology exposure in medical schools [11-13]. Fewer medical schools with urology residencies likely result in diminished mentorship opportunities and reduced access to original research and scholarly activities. Studies have shown that exposure to a specialty and mentorship significantly influence students' career choices [14,15]. Medical schools with larger urology programs and more faculty tend to match more students in this specialty [16]. Similarly, matched applicants and those from top NIH-funded schools often have higher publication counts, with applicants to the top 50 urology programs averaging two PubMed-indexed urology papers, including at least one first-author paper [17,18]. Publication numbers positively correlate with match success, underscoring the need to address differences in research and mentorship opportunities for aspiring urologists [19]. Distinctively expanding on the current literature, our study adds new insights by highlighting significant growth in medical school numbers established after 2000 and a correlating decline in the proportion of urology residency positions offered. Additionally, we present evidence of varying NIH funding levels across AUA sections to highlight the regional impacts of the rise in medical schools, further emphasizing the barriers created for urology residency applicants.

This study has limitations. Not all medical schools had publicly available data at the time of analysis. Additionally, NIH and BRIMR database figures may not precisely reflect funds allocated to specific medical schools but rather the overall NIH support received by grantees. Despite these limitations, BRIMR rankings are widely used as indicators of scientific vitality [9]. While the study demonstrates a strong association between urology residency presence and NIH funding, it does not establish causality. Factors such as institutional prestige and faculty numbers likely play a role and warrant further investigation.

This study did not include qualitative perspectives from stakeholders, including students, faculty, and program directors. Future research incorporating interviews or surveys could offer deeper insights into aspiring urologists' challenges and provide targeted recommendations for expanding research and mentorship opportunities.

## Conclusions

The increasing proliferation of medical schools relative to urology residency programs presents a potential hurdle for prospective urology candidates. Recognizing the significance of research and mentorship, it is imperative to establish accessible programs at both the AUA section and national levels for students interested in applying for a urology residency. Specifically, we found that students at schools without urology residency programs face unique challenges, and we recommend implementing urology-focused research fellowships and offering clinical rotations at hospitals with urology residency programs to enhance these students' experience and competitiveness in the residency match.
